# Understanding Active Photoprotection: DNA-Repair Enzymes and Antioxidants

**DOI:** 10.3390/life14070822

**Published:** 2024-06-28

**Authors:** Emilio Garcia-Mouronte, Luis Alfonso Pérez-González, Jorge Naharro-Rodriguez, Montserrat Fernández Guarino

**Affiliations:** Dermatology Department, Hospital Universitario Ramon y Cajal, Carretera M-607 km 9.1, 28034 Madrid, Spain; pg.l.alfonso@gmail.com (L.A.P.-G.); montsefernandezguarino@gmail.com (M.F.G.)

**Keywords:** DNA damage, sunscreening agents, DNA-repair enzymes, antioxidants, actinic keratosis, skin neoplasms

## Abstract

The detrimental effects of ultraviolet radiation (UVR) on human skin are well-documented, encompassing DNA damage, oxidative stress, and an increased risk of carcinogenesis. Conventional photoprotective measures predominantly rely on filters, which scatter or absorb UV radiation, yet fail to address the cellular damage incurred post-exposure. To fill this gap, antioxidant molecules and DNA–repair enzymes have been extensively researched, offering a paradigm shift towards active photoprotection capable of both preventing and reversing UV–induced damage. In the current review, we focused on “active photoprotection”, assessing the state-of-the-art, latest advancements and scientific data from clinical trials and in vivo models concerning the use of DNA-repair enzymes and naturally occurring antioxidant molecules.

## 1. Introduction

### 1.1. Photocarcinogenesis

Photocarcinogenesis is a complex process where ultraviolet (UV) radiation from sunlight contributes to skin cancer development through various molecular mechanisms [1]. UV radiation (UVR) includes the UVA, UVB, and UVC bands, with UVA and UVB being the most significant in sunlight exposure. These radiation types penetrate the skin, inducing DNA damage directly or indirectly, leading to mutations that can result in skin cancer [1].

UVR increases the production of reactive oxygen species (ROS), which can damage DNA, proteins, and lipids in skin cells, contributing to carcinogenesis [2]. The interaction between UV light and the skin activates specific signaling pathways that lead to DNA mutations [3]. Prolonged UV exposure can cause chronic inflammation, which promotes tumor growth and invasion by acting in the tumor microenvironment, promoting angiogenesis, and suppressing the immune response to tumor cells [2]. Key molecular players in photocarcinogenesis include the p53 protein, a tumor suppressor gene that plays the main role in controlling cell cycle arrest and apoptosis in response to DNA damage [2]. UV-induced mutations in the p53 gene are common in skin cancers, leading to the loss of function of this critical protein, allowing uncontrolled cell division [3]. Another mechanism involves the activation of the MAP kinase pathways, which influences cell proliferation and survival, further contributing to the development of skin cancer [4]. Additionally, the upregulation of RAS oncogenes and the inactivation of tumor suppressor genes, as PTEN, are also pivotal in the development of skin cancer following UV exposure [4]. These genetic changes accumulate over time, increasing the risk of malignant transformation of skin cells [4]. Research continues to uncover the detailed molecular pathways involved in photocarcinogenesis, offering potential targets for prevention and treatment strategies [1,3]. 

### 1.2. Photoaging and Hyperpigmentation

Photoaging is characterized by deep wrinkles, hyperpigmentation, and loss of skin elasticity and results from chronic exposure to UV [5]. Both UVA and UVB damage the skin through the generation of ROS, which disrupt DNA and degrade collagen, accelerating the aging process [5]. UVA rays penetrate deeper into the dermis, exacerbating photoaging by breaking down collagen and elastin, the fibers that support skin structure [5]. UVB rays, though primarily affecting the epidermis, also contribute significantly to photoaging by promoting the formation of wrinkles and increasing epidermal disorganization, thickness, and roughness [5]. The oxidative stress from UV exposure leads to the inflammation and degradation of collagen and elastic fibers, which are essential for maintaining skin’s youthful appearance [6]. Preventive measures against photoaging include the application of broad-spectrum sunscreens (SC) that protect against both UVA and UVB [6]. Modern formulations often include antioxidants and other additives that not only protect against but may also reverse some signs of aging [6]. 

Industrialization, digitalization, and the fast-growing use of electronic devices such as mobile phones, laptops, and tablets haved had a significant impact on daily lifestyle, with increased and continuous exposure to artifical light sources, including blue light [7]. In vitro studies have shown blue light exposure leads to DNA damage, oxidative stress alteration of collagen and elastin, reduced immunosurveillance, and increased apoptosis [7,8]. A dose-dependent influence has been suggested [8]. Additionally, what is clearly demonstrated among studies of blue light on the skin is the induction of hyperpigmentation [8,9].

Thus, the recognition that visible light (VL) and infrared radiation contribute to photoaging suggests the need for SCs that cover a broader spectrum of light [6]. The use of tinted SCs, which combine physical blockers like zinc oxide and titanium dioxide with iron oxides, can protect against the full spectrum of light contributing to photoaging [10].

Photoprotection is essential for safeguarding the skin from the harmful effects of electromagnetic radiation, particularly UV and VL [1]. There are various types of photoprotection strategies available [1]. These include physical measures such as wearing photoprotective clothing, seeking shade, and using broad-spectrum SC [1]. Broad-spectrum SCs are critical as they provide protection against both UVA and UVB rays, the main contributors to skin damage [6]. In terms of SC composition, there are both organic and inorganic filters [6]. Moreover, tinted SCs provide an additional layer of protection against VL, which can contribute to photoaging and hyperpigmented conditions like melasma [10]. 

### 1.3. Active Photoprotection

Photoprotection encompasses a wide set of behavioral, physical, and chemical measures aiming at preventing or limiting the acute and chronic deleterious effects of UVR on human skin [11]. Although avoidance of sun exposure is by far the most effective photostrategy, its real-life application is extremely challenging and often impracticable, especially in outdoor workplaces and regions with high solar radiation [12,13]. 

SCs are composed of mineral and organic compounds (filters) capable of scattering and neutralizing different wavelengths, including UVR and VL [12]. Due to their ease of application, continuous pharmacological innovation, and consciousness-raising awareness, their use has become widespread in numerous geographic areas during the last several decades [12]. 

Depending on their mechanism of action, SCs can be subdivided into “passive” and “active” [12]. Passive photoprotection, which is the traditional and most frequently employed approach, consists in the topical use of filters that reflect UVR and VL [12]. Despite their mechanism of action, they can indeed be overcome by inflicting radiation, especially when inappropriately applied [12]. Furthermore, filters are ineffective in reverting skin damage induced by UVR and VL once it has happened [12]. To fill this gap, chemical compounds have been extensively researched to limit the oxidative insult and DNA damage after sun exposure, which are collectively known as “active photoprotection” [12]. Most of these substances are naturally occurring molecules extracted from plants and algae [13]. Despite their recent development and increasing use, scientific reviews assessing the efficacy and safety of active photoprotective agents as a whole are lacking in the available literature. Available reviews on this topic are scarce, are mostly focused on a certain subset of agents, and consist mainly of preclinical or animal studies [12,13,14]. 

For these reasons, the aim of this work is reviewing the state-of-the-art in active photoprotection by thoroughly assessing the current scientific available data on the use of antioxidant substances and DNA-repair enzymes in human skin, especially in in vivo models and clinical trials. 

## 2. DNA Repair Enzymes

### 2.1. Photolyases

#### 2.1.1. Evolutionary Origin and Mechanism of Action

UV exposure may result in notable deleterious effects on human skin cells. Although single and double strand-breaks can occur, most changes in chromosomal DNA consist of chemical modifications of nitrogenous bases, especially cyclobutane pyrimidine dimers (CPD) and pyrimidine (6-4) pyrimidone photoproducts (6-4PP) [15,16,17]. When interfering with key cellular processes such as replication and transcription, they lead to cell cycle arrest and ultimately apoptosis [18,19]. Conversely, if DNA damage is persistent, numerous gene mutations can build up, allowing cells to escape from controlled growth and increasing the risk of malignant transformation [18].

For these reasons, organisms have developed during the course of time a complex network of repair mechanisms with different but complementary substrates, which keep DNA under constant monitoring [20]. The removal of photolesions is mostly conducted by the highly evolutionary conserved nucleotide excision repair (NER) pathway [20]. While this system rapidly corrects common photoproducts, such as 6-4PPs, its effects on CPD are far more modest [21]. 

A wide array of organisms found in the archaea, bacteria, and eukarya domains have mounted an additional DNA-repair mechanism known as photoreactivation, firstly described by Kelner in 1949 [18,22,23,24,25]. This is based on photolyases, flavoprotein-based monomeric enzymes that repair damaged ssDNA and dsDNA using blue wavelength as an energy source [16,22,23,26]. Compared to NER, the biochemical mechanism is more simple and consists of the rapid cleavage of photoproducts into their original undamaged state, thanks to electron transfers from the photo-excited flavin to the dimer [18,23,27]. Interestingly, photolyases show marked substrate specificity and are subdivided into CPD photolyases and 6-4PP photolyases [28,29].

Nevertheless, placental mammals, including humans, are strictly dependent on the NER pathway to repair UV-induced DNA damage [19]. According to the “nocturnal bottleneck hypothesis” conjectured by Menaker et al. [30], early eutherian mammals were small night-active insectivores living on trees [31]. Arising in the Mesozoic era after the Permian–Triassic event, they were forced to live in light-restricted environments as they faced inter-species competition and predation pressure by diurnal dinosaurs [31]. These factors led to significant changes in their homeostasis, such as the evolution of endothermia, the loss of underused photoreceptor structures and certain photoprotective mechanisms such as photolyases in light-exposed tissues [32]. 

#### 2.1.2. Scientific Evidence: General Overview

The use of topical photolyases (TPHs) as active SCs is overwhelmingly supported by current data [33,34,35,36,37,38,39,40,41,42,43,44,45,46,47,48]. Since 2000, four randomized control trials (RCTs) [35,42,44,47], one experimental trial [33], five pilot interventional studies [34,37,38,39,45], one longitudinal observational clinical trial [41], and five observational studies [36,40,43,46,48] have been published that underline the efficacy of TPHs in the management of actinic keratoses (AKs) (n = 12) [36,37,38,39,41,42,43,44,45,46,47,48], but also in additional disorders such as *xeroderma pigmentosum* (XP) [40], polymorphic light eruption (PoLE) [35], and in the general population [33,34]. In almost a half of these works (n = 7), the use of TPHs was compared against regular SPF30-50+ SC [34,35,39,42,44,45,47]. Although the follow-up periods were heterogeneous (3 days [33]–1 year [40]), most of the studies (n = 9) [37,40,41,42,43,44,45,46,48] were long enough (≥3 months) to validly assess the clearance of AK lesions. 

Different and complex methods have been employed to study in vivo the effects of TPHs on human skin, including clinical scores (AKASI, AKSS, FPS) [47], non-invasive diagnostic techniques such as dermoscopy [39,41,45], reflectance confocal microscopy (RCM) [35,41,45], fluorescence [42], colorimetry [37], telethermography [43], biopsies [33,34,38,39,40,42], and RNA extraction [38]. 

Regarding the source of TPHs, all authors conducted their investigations with photolyases (0.5–1%) extracted from the blue–green algae *Anacystis nidulans* and encapsulated in liposomes [33,34,35,36,37,38,39,40,41,42,43,44,45,46,47,48]. These are mostly commercialized under the mark Eryfotona AK-NMSC fluid^®^ (Isdin, Barcelona, Spain), which is a film-forming medical broad-spectrum SPF100+ SC. In two works [35,42], patients were simultaneously treated with topical endonucleases (0.5–1%) derived from the bacterium *Micrococcus luteus*. Most were combined with broad-spectrum SPF50+ SC [34,36,37,38,39,40,41,42,43,44,45,46,47,48]. Depending on the clinical indication, the TPHs were applied 30 min before UVR [34], 5 [35]–60 min [33] post–UVR, once [41] or twice daily [36,38,42,43,44,45,46,47,48]. 

#### 2.1.3. In Vivo Pilot Experimental Trials 

The first in vivo experimental trial that confirmed the reduction of CPD levels in human skin by TPHs was published by Stege et al. [33] in 2000 (Table 1). Buttock sites (area = 16 cm^2^) of 19 healthy participants with low phototypes (II–III) were exposed to UVB radiation (1–2 minimal erythema doses (MEDs)) and treated afterwards with a hydrogel containing Anacystis nidulans 1% encapsulated in liposomes [33]. Differently from commercialized products, the hydrogel lacked filters, which allowed the authors to assess the effects of TPHs on irradiated human cells, avoiding the synergistic influence of SC [33]. The photoactivation of TPH was achieved using blue light (λ = 340–450 nm) for 30 min [33]. Biopsies were taken immediately after and 1 day after UV exposure [33]. CPD levels were assessed by fluorescence [33]. UVB (1 MED) significantly increased the presence of CPD in human cells compared to sham-irradiated sites (97 au vs. 10 au) [33]. After photoactivation, TPH rapidly reduced the levels of CPD in a time-dependent manner (1 min: 100 au, 5 min: 87 au, 30 min: 60 au, −42%). The repair effect was at its maximum level just after the completion of photoactivation, since the amount of CPD did not change 24 h after TPH administration (−44%). The authors also investigated whether TPH could partially revert UV-induced immunosuppression [33]. The expression of ICAM-1, a protein vital for eliciting cellular immune responses, was assessed before and after the subcutaneous administration of IFN-γ (3800 UI) through fluorescence. ICAM-1 levels in sham-irradiated areas and UV-radiated did not change after IFN-γ administration (10 au vs. 8 au and 52 vs. 55 au, respectively) [33]. TPH managed to partially decrease its expression after IFN-γ injection and photoactivation (29 au) [33]. 

Eleven years later, Berardesca et al. [34] published the results of a pilot interventional clinical study comparing for the first time the use of TPHs combined with broad-spectrum SPF50+ SC to SC alone (Table 1) [34]. Ten healthy young individuals with FFII were recruited [34]. Five experimental sites from unspecified anatomical locations were used as the control (n = 1) and exposed to ssUVR, ssUVR + Vehicle, ssUVR + SC and ssUVR + TPH + SC [34]. ssUVR (λ = 290–400 nm, 3 MEDs) was administered daily for 4 consecutive days [34]. Topical products were applied once daily, 30 min before each ssUVR [34]. Biopsies were taken from all sites 3 days after the last UV exposure [34]. ELISA was employed for CPD determination [34]. Compared to the baseline, the expression of CPD dramatically increased after ssUVR exposure (x19) and was not mitigated by the application of the vehicle (x19) [34]. TPH + SC significantly reduced the levels of CPD after ssUVR (−93% compared to ssUVR-sites) and were superior to SC alone (−62%, *p* < 0.001) [34]. However, no treatment managed to revert the amount of CPD in human skin to baseline levels before ssUVR [34]. In addition to preventing the formation of photoproducts, TPH + SC considerably mitigated UVR-induced DNA fragmentation and cellular apoptosis (−82%) and were again superior to SC alone (−40%, *p* < 0.001) [34]. These results indicated TPHs could be employed as an additional constituent in broad-spectrum SC, palliating the deleterious effects of UVR not appropriately neutralized by filters [34]. Since no treatment fully reverted DNA damage to baseline levels, sun avoidance continues to be the best photoprotective strategy [34].

#### 2.1.4. Actinic Keratoses

Concerning the use of TPHs on AKs, the recruited participants were mostly middle-aged and elderly Caucasian males (40–100%, 28–92 years) [36,37,38,39,41,42,43,44,45,46,47,48] with FFII-III [36,37,39,40,43,44,47,48]. Interestingly, some of the studies even included high-risk individuals such as patients with XP [39,40] and those with a personal history of non-melanoma skin cancer (NMSC) [36,39,40] or immunosuppression [39,46]. As these commonly show severe cancerization fields and accumulate great amounts of UV-induced DNA mutations, they could benefit significantly from the use of TPHs [49]. The scalp and face were the most commonly involved sites [36,37,41,42,43,44,45,48].

Assessing therapeutic efficacy for AK is extremely challenging, both in clinical trials and in the real-life setting [50]. Efficacy is assessed in most studies based on the count of macroscopic lesions by the investigators, with clear inter-observer variation. Additionally, results are usually presented using clearance rates, which vary across studies. These may include the reduction in AK count, percentage of patients achieving clerance greater than 50%, complete clearance, and incidence of new AK lesions… Comparison of the efficacy of a treatment between studies can thus be extremely difficult. 

Following this strategy is suboptimal, as it does not take into account important factors such as the severity of mutations, epithelial dysplasia, erythema, and roughness in the “normal” skin (field cancerization) surrounding the clinical lesions [51]. Several authors have tried to overcome these limitations by assessing the efficacy of treatments in biopsies and using histological and genetic parameters [52]. However, samples are either taken from randomly selected lesions or from those considered to be the most severe [38]. The latter is an inappropriate method, as Schmitz et al. [53] reported that physician-perceived severity (i.e., Olsen score) does not accurately match histological findings and the risk of transformation into squamous cell carcinoma (SCC). 

The natural evolution of macroscopic lesions indeed shows extreme inter- and intraindividual variability [54]. On the one hand, the spontaneous resolution of AK lesions is a well-known phenomenon, with an estimated annual rate of 25% [54]. On the other hand, the risk of malignant transformation into invasive SCC is not negligible. Lee et al. [55] recently estimated the hazard ratio in AK patients 80 years or older for developing SCC at 5.69. For these reasons, different clinical (i.e., AKASI) and histological scores (i.e., PRO) were designed to better predict the risk of malignant transformation [51,52]. However, these tools are not exempt from limitations that hinder their widespread use in the real-life setting. 

The previously mentioned limitations hindered comparisons between studies and the calculation of a global clearance rate encompassing all TPH trials. Despite this, TPHs have been shown to be an excellent active treatment, reducing the count of AK lesions [36]. Clearance rates 50% or greater were present in 20 [47]–100% [36,46,48] of the treated patients, while 29 [48]–42.86% [38] of the individuals exposed to TPHs achieved complete resolution of the lesions. Assessing the available results as a whole, most participants managed with TPHs achieved at least some degree of improvement greater than the rate of spontaneous resolution of AK lesions [36,37,38,39,40,44,45,46,47,48]. Reduced incidence of new lesions has also been reported in three studies [40,45,48].

Improvement in other clinical parameters beyond the clearance of AK lesions has also been observed. After TPH exposure, field cancerization and AK lesions showed less erythema [39,41,48], scaling [39,41,48], and pigmentation [48], assessed both clinically and by dermoscopy. 

TPHs maintain a proper long-term clearance of AK lesions after previous photodynamic therapy (PDT) with methyl aminolevulinic acid (MAL-PDT) (λ = 630 nm, 37 J/cm^2^) [44]. A total of 30 patients with face or scalp AKs were randomly assigned to receive Eryfotona AK-NMSC fluid^®^ (*Anacystis nidulans* encapsulated in liposomes) or standard SC (1:1), twice daily for 9 months (Table 2) [44]. The basal AK count was relatively low (2 ± 2 vs. 0.6 ± 0.5, *p* > 0.05), as the last MAL-PDT treatment was administered 2 weeks before the administration of Eryfotona^®^ [44]. At the final visit, the TPH-exposed patients showed lower AK counts (1 ± 1.1 vs. 3.6 ± 3.8, *p* < 0.01) and a reduced need of new PDT sessions (0 vs. 10, *p* = 0.01) [44]. Most (87%) SC-treated patients developed at least one new AK lesion during the trial [44]. The application of these findings in real-life practice is crucial, as this research clearly indicates TPHs achieve an excellent long-term clearance of AK lesions [44]. 

Recently, a double-blinded RCT assessed the efficacy of TPHs in AK sited on the forearms (Table 2) [47]. Although previous studies had included patients with AKs in this anatomical region [39,46], none had specifically addressed this location [47]. A total of 40 AK patients were randomly assigned to treatment courses of TPH + SC SPF99 vs. SPF99 alone (1:1) twice daily for 2 months [47]. The recruited participants were mostly women (60%) with FFII-III [47]. The baseline field-cancerization severity was at least moderate, with a baseline lesion count on each forearm of 7 (6–9), a forearm AK severity score (AKSS) of 72 (51–95) and forearm photoaging scale (FPS) of 107 (91–116) [47]. AK count reduction was similar in both groups after completion of the treatment (5 vs. 5, −38.7%) [47]. Clearance rates were modest, as few patients managed to achieve a reduction in the number of AK lesions greater than 50% (25% vs. 20%) [47]. The final AKSS (38 vs. 32) and FPS (78 vs. 85) scores were inferior to baseline levels, although no statistically significant differences were found between groups [47]. One patient in each group developed one NMSC during the trial [47]. The TPHs failed, thus, to add any benefit to standard SC in the treatment of AKs located on the forearms, which is precisely one of the sites most challenging to manage [50].

Non-invasive diagnostic techniques have shed light on the effects on tissular and cellular structure after the topical application of photolyases (Table 3). 

Puig et al. [39] directed the first controlled interventional clinical study which robustly assessed the efficacy of TPHs in AK management using dermoscopy and RCM (Table 3) [39]. Eryfotona AK-NMSC fluid^®^ and SPF50+ SC were independently used twice daily for 1 month in 11 low-phototype patients (3:1) with AKs on the head, scalp, and forearms [39]. Most participants were at high risk: eight (61.54%) had a personal history of NMSC, two were affected by XP (15.38%), and one was under immunosuppression (7.69%) [39]. Only patients treated with TPH achieved complete (44.44%) or partial (33.33%) clearance of AK lesions at the end of the study [39]. Dermoscopic evaluation showed improvement in erythema (*p* = 0.03) and scaling (*p* = 0.028), without changes in pigmentation or follicular plugs (Table 3) [39]. After TPH treatment, RCM improved in several items compared to the baseline, with significantly less scaling (−78.81%, *p* = 0.004), fewer round cells at the stratum granulosum (−57.14%, *p* = 0.019), and less atypical honeycomb pattern (−67.13%, *p* < 0.0005), as well as a decreased coherence of corneocytes (+72%, *p* = 0.018). No changes were detected in the density of nucleated cells in the stratum corneum (*p* = 0.221), disarray of epidermal pattern (*p* = 0.095), vascularization (*p* = 0.413), and inflammation (*p* = 0.221) [39]. Interestingly, no improvement in dermoscopy or RCM was detected in the three SC-treated individuals (Table 3) [39]. 

These RCM findings were not later replicated by Moscarella et al. [45], as no statistically significant difference was detected between TPH and SPF50+ SC after 6 months of treatment (Table 3). This study had a larger sample size (n = 50 vs. n = 13), a longer follow-up period (6 months vs. 1 month) and the second-highest AK basal count (13 ± 6.8) of all of the published works [39,45]. Most cases were severe, as 60% (n = 30) of the participants had more than 10 AK clinical lesions within the target area [45]. At the end of the treatment, the AK count (−3.8 vs. −3.3) and investigator-assessed severity (−0.2 vs. −0.2) dropped in both groups, although no statistical analysis of the results was shown [45]. No differences (1.2 vs. 1) were found in the investigator global improvement index (range: −2 vs. +7) [45]. TPH-treated patients had higher baseline AK counts (13 ± 6.8 vs. 10.9 ± 0.5), which could explain why photolyases only proved to be superior after the subgroup analysis, specifically preventing the appearance of new AK lesions in mild cancerization fields (0.1 vs. 1.5, *p* = 0.04) [45].

Rstom et al. [41] confirmed the positive evolution of facial AKs on dermoscopy after TPH treatment (Table 3) [41]. Fourteen grade I and three grade II AK lesions were treated with Eryfotona AK-NMSC fluid^®^, once daily for 3 months [41]. After completing their administration, 64.29% of the grade I lesions experienced at least some degree of improvement in both erythema and scaling, with a partial normalization of the atypical honeycomb pattern with RCM [41]. However, dermoscopy and RCM failed to detect any improvement in grade II lesions (Table 3) [41]. Increased thickness of the epidermis may reduce the percutaneous absorption of TPHs in vivo and thus minimize their concentration in the cell nuclei.

As previously mentioned, different groups of investigators have evaluated the effect of TPHs on human skin through different optical devices such as fluorescence, spectrophotometry, or telethermography (Table 3) [35,37,42,43]. In 2015, Laino et al. [43] published the results of a prospective cohort of 30 low-phototype AK scalp patients treated with TPH, twice daily for 9 months [43]. Efficacy was assessed through active teletermography (ATT) [43]. ATT analyzes the vascular pattern of tumors by detecting the infrared radiation emitted from a lesion before and after the rapid removal of a thermal stimulus [43]. Results were expressed in the form of thermal recovery time (TRT) and subdivided into three categories: rapid (R: <10 s), slow (S: 10 s–2 min), and normal (N: >2 min) [43]. Before the onset of the TPHs, all AK lesions had a rapid TRT (100%) [43]. At the 9th month of treatment, TPHs managed to increase the TRT; most lesions showed a slow TRT (n = 19, 70.37%) and even in 3 AK (11.11%) was completely normalised [43]. On the other hand, at baseline evaluation, all AK lesions (100%) displayed a surrounding hyperthermic halo (HH), whose overall size considerably diminished after treatment (0.61 cm^2^ vs. 3.45 cm^2^, −82.37%) [43]. These outcomes suggested TPHs could reduce the hypervascularization associated with skin carcinogenesis [43]. Puviani et al. [37] reached a similar conclusion in the same year. Target areas from 11 AK patients with low phototypes (II-III) were treated with Eryfotona AK-NMSC fluid^®^ twice daily for 3 months [37]. Anatomical location was not detailed, although most lesions were sited on the face and the scalp [37]. Colorimetry was chosen to evaluate the clinical response [37]. This procedure is based on a scanning device that assesses changes over time in melanin, hemoglobin, and skin profiles [37]. Content of hemoglobin partially diminished in the lesions (−34%, *p* = 0.0125) where TPHs were applied [37]. Additionally, the size of the target lesions dramatically decreased after TPH treatment (−75%, *p* < 0.01) [37].

Overall improvement in field cancerization has been demonstrated in an RCT (Table 3) [42]. Caucasian patients (n = 28) with thin (grade I and II) facial and scalp AKs were randomly assigned to receive treatment with a combination of photolyases (1%, *A. nidulans*), endonucleases (1%, *M. luteus*), and SPF50+ SC vs. SC alone [42]. The products were applied twice daily for 6 months [42]. The photodamage was moderate, as the AK count at onset ranged from 4 to 10 in each target area [42]. Topical application of methyl aminolevulinate acid (MAL) was used to assess the fluorescence of field cancerization [42]. An incubation period of 3 h was followed [42]. At the 6th month visit, the combination was superior to SC alone (Table 3) in reducing the field cancerization fluorescence (−29.09% vs. −10.19%, *p* < 0.001) and CPD levels (−61.76% vs. −34.48%, *p* < 0.001). 

Puig-Batillé et al. [38] conducted the first and only clinical trial to this date which assessed the effects of photolyases on the expression of different genes in human skin in vivo [38]. Eleven AK patients were treated with Eryfotona AK-NMSC fluid^®^ twice daily for 1 month. Biopsies were taken before (T0) and after (T1) the completion of the treatment [38]. Complete responders showed a significantly lower final expression of TNF (*p* = 0.012) and upregulated levels of WDR72 (*p* = 0.04) and CPI-17 (*p* = 0.039) [38]. CPI-17 expression was higher than in the partial responders (*p* = 0.0001) [38]. The authors concluded that increased expression of CPI-17 controlled MYPT1-PPδ activity, regulating the dephosphorylation of the retinoblastoma protein and subsequently improving cell cycle control and adhesion [38]. However, the final sample size was low, as 4 out of 11 patients (36.36%) refused to take a second biopsy due to personal issues, or the RNA extraction failed [38]. Puig et al. [39] later confirmed that TPHs regulate the cell cycle, as keratinocyte expression of p21 decreased after 1 month of treatment (−38.71%, *p* = 0.042).

Giustini et al. [40] particularly evaluated the use of TPH as a photoprotective agent in the real-life management of eight patients with XP (Table 2). XP-affected individuals suffer from different genetic defects in the DNA repair system, making them extremely vulnerable to UV-induced skin carcinogenesis [56]. Apart from their preventive properties, TPHs could serve as an excellent therapy in this disease, actively reducing the accumulation of CPD dimers and the risk of malignant transformation [22]. The recruited patients were middle-aged (55) with previous long exposure to solar UV radiation (sUVR) [47]. During the last year before TPH application, a mean of 6.8 basal cell carcinomas (BCC) and 3 SCC had been diagnosed [40]. Eryfotona AK-NMSC fluid^®^ was prescribed twice daily for a year [40]. The product achieved a significant reduction in the appearance of new AK lesions compared to the year before (5, −64.29%) [40]. Remarkably, this has been the only research that hinted at the possibility that TPH reduces practice the development of BCC (n = 3, −56%) and SCC (0, −100%) in real life [40]. As a result, the use of TPHs and SCs must be thoroughly encouraged in XP patients [40]. Further studies are warranted to confirm the potential benefits of long-term administration of TPHs in the quality of life and long-term survival of XP patients [40].

Beyond clinical trial data, three observational studies have confirmed the benefits of TPH in real-life AK patients (Table 4) [36,46,48]. 

Vañó Galván et al. [48] published in 2016 the largest open-label longitudinal prospective study (n = 41). A total of 41 patients with ≥4 AKs, all treatable with cryotherapy, were included (Table 4) [48]. Participants were excluded if they had received field-cancerization-targeted treatments in the previous 3–6 months [48]. Most were male (n = 37, 90.24%), with a mean age of 75.3 years (58–85) and FFII [48]. AK lesions were mainly located on the scalp (n = 33, 80%). Eryfotona AK-NMSC fluid^®^ was started 1 day after cryotherapy and continued twice daily for 6 months [48]. Nonetheless, this work has some biases that limit the validity of its outcomes and conclusions. Firstly, a specific definition of AKs treatable with cryotherapy was lacking [48]. Since the global baseline number of AK lesions was high (9.56), this indicates that patients with even higher basal counts were nevertheless included [48]. Furthermore, no details were given of why the investigators prioritised destructive therapies over field cancerization therapies precisely in this subset of severely photodamaged participants [48]. Secondly, efficacy was assessed strictly by clinical means, with the obvious limitations previously mentioned [48]. As there was a lack of a comparator, the final outcomes cannot be fully attributed to the use of Eryfotona AK-NMSC fluid^®^, as several patients were continuously and discretionally exposed to adjuvant cryotherapy while receiving treatment [48]. In this respect, the percentage of patients that required this therapy while on TPHs is not detailed [48]. Therefore, the percentage of participants that achieved partial or complete clearance (100% and 29%) is thus overestimated, as lesions refractory to TPHs could have been precisely destroyed by cryotherapy [48]. Consequently, three groups of patients (TPH vs. cryotherapy alone vs. cryotherapy + TBP) would have been necessary to correctly discern the overall efficacy, tolerability, and safety of TPH [48]. 

Smaller clinical series (n = 6–9) were published by Puviani et al. [36] and Navarrete-Dechent et al. [46]. AK patients mainly on the face and scalp were treated with Eryfotona AK-NMSC fluid^®^ twice daily for 1.5 [36] to 3 months [46]. Almost all of the patients were middle-aged to elderly males [36,46]. Participants with a personal history of NMSC and immunosuppression were also recruited [36,46]. All patients in both series achieved a clearance of AK lesions ≥50% after completing the treatment (Table 4) [36,46]. No remarkable side effects were identified [36,46].

Self-perceived local tolerability and sense of improvement are important factors for achieving therapeutic success in chronic disorders such as AK [50]. Both were highly rated by patients in the study conducted by Vañó Galván et al. [48], with mean scores of 2.71 and 2.85, respectively (range: 0–4). After 6 months of treatment, patients’ mean adherence was remarkably high, with a score of 3.21 (range: 0–4) [48]. In the trial conducted by Moscarella et al. [45], patients’ satisfaction with treatment (5, range of the score 0–7) and assessment of local tolerability (5.1, range of the score 0–7) were remarkably good. These outcomes underline the ease of use of TPH and could indicate proper compliance in the real-life setting. 

Overall tolerance of TPH is considered excellent, which is an important aspect of chronically prescribing a twice-daily topical product [35,36,37,40,43,44,45,47,48]. The rate of adverse reactions was low (0 [35,48]–7.5% [45]). No severe side effects warranting discontinuation of the treatment were detected [35,36,37,40,43,44,45,47,48]. 

Despite the previously mentioned benefits, the outcomes are not exempt from biases and limitations that partially limit their validity. Firstly, the sample size was modest (n < 50) in all of the experimental trials and observational studies [33,34,35,36,37,38,39,40,41,42,43,44,45,46,47,48]. Mean baseline AK count considerably varied between studies, which hampers a proper comparison and extrapolation of the results [40,41,44]. On the other hand, most authors did not specify further details crucial to infer the field cancerization severity, such as the personal history of on-site NMSC, previously prescribed treatments, or affected surface area [36,38,39,40,41,43,45]. Additionally, only in five studies were patients excluded if they had received field-cancerization-targeted treatments 3–6 months before the use of TPHs [42,46,47,48]. Persisting benefits of recently prescribed therapies, such as imiquimod, may increase the clearance of AK lesions up to 6 months after completion, masking any potential differences of TPH compared to the placebo or standard SC [57]. 

In addition to the latter, one of the greatest limitations of these studies is the still unknown effect of TPHs on skin carcinogenesis. Until the date of publication of this review, no work has specifically addressed the incidence of skin tumors in patients chronically treated with TPH. This is obviously crucial in the clinical setting, as the main aim of treating AKs is precisely reducing the risk of malignant transformation. Proper well-designed RCTs with larger sample sizes and longer follow-up periods are thus necessary for assessing this hypothesis. Future research warrants the development of clinical and histological scores with high inter- and intraindividual correlation for accurately assessing the efficacy of TPHs in AK patients and enabling an adequate comparison between studies. 

#### 2.1.5. Polymorphic Light Eruption 

Although most research on the use of TPHs has been centred on the management of AKs, photolyases could prove beneficial in photosensitive disorders such as PoLE [35]. In a randomized double-blinded placebo-controlled intra-individual half body trial, topical combined photolyases and endonucleases (without filters) were compared to SPF30 SC and a placebo in 14 patients with PoLE and FFII-III [35]. This RCT is interesting in different aspects, but especially in its recruitment of individuals with photosensitive disorders, which were excluded in the remaining studies (except XP patients) [33,34,36,41,42,44,47]. Two pairs of symmetrically distributed test fields (5 × 5 cm) in PoLE-predilection sites were selected for photoprovocation, which was performed once daily for 4 consecutive days. Sites were allocated to receive no treatment, SPF 30 SC (20 min before each UVR), TPHs (5 min after photoprovocation), and a placebo (5 min after provocation) [35]. Authors employed a new clinical tool for evaluating the response (range: 0–12), assessing items such as percentage of site area affected, infiltration, and pruritus [35]. SPF30 SC proved to be superior to TPHs, with a lower PLE score (*p* = 0.00391), erythema, and pigmentation measured through reflectance spectroscopy (*p* = 0.00012 and *p* = 0.00037, respectively) [35]. Lesions were nearly absent in sites pretreated with SPF30 SC [35]. Compared to the placebo, TPHs only managed to be superior in terms of pruritus (1.73 vs. 3.2, −46%, *p* = 0.02441). Avoiding or filtering UV photons are thus the best methods for preventing the elicitation of PoLE lesions. However, it should not be forgotten that the mechanism of action of TPHs consists mainly in the breakdown of photoproducts, which accumulate precisely after the UV non-filtered radiation has taken place. For these reasons, we consider that it could be interesting to discern whether the use of TPHs might have a synergistic effect in the management of PoLE when combined with SC, as they could reduce the formation of neoantigens and thus break the initial trigger of the pathogenic chain. 

### 2.2. T4 Endonuclease V

#### 2.2.1. Biological Origin

Tanaka and his team [57] discovered in 1975 that bacteriophage T4 endonuclease V (T4N5)- a 16,500-Da polypeptide from *Escherichia coli* infected with bacteriophage T4-, could enhance nucleotide excision repair in human cells and initiate the removal of CPD [58,59]. T4N5 production is regulated by the v+ gene of T4. For this reason, extracts from uninfected *Escherichia coli* showed minimal function. Interestingly, T4 mutants with a higher sensitivity to UVR did not display any enhanced enzymatic activity [60]. Later, in the 1980s, it was found that this enzyme could be delivered to cells using liposomes, which are microscopic spheres made up of lipid bilayers that form spontaneously in water, stabilizing the compound, slowing its transit through the skin, and improving percutaneous absorption [61]. T4N5 liposomes have proven effective in delivering repair enzymes to cultured cells [62]. 

#### 2.2.2. Mechanism of Action

T4 endonuclease plays a crucial role in initiating DNA repair at sites of UV-induced CPDs, which, if left unrepaired, can lead to mutations causing non-melanoma skin cancer [63]. The enzyme binds to non-target DNA in a salt-dependent manner and utilizes facilitated diffusion to locate its target site [64]. Upon detecting UV-damaged DNA, cleavage occurs through two combined activities, the pyrimidine dimer–DNA glycosylase activity and the apurinic–apyrimidinic endonuclease activity [65]. This enzyme enhances the natural DNA repair process approximately fourfold [59]. Additionally, T4 endonuclease V promotes skin regeneration and reconstruction, preventing the breakdown of extracellular matrix components and thereby helping to combat photoaging [66]. For example, treatment with T4N5 reduces MMP-1 induction in human skin cells similarly to photolyase treatment, resulting in decreased collagen degradation [66]. The single polypeptide of T4N5 can substitute for the human multi-enzyme complex to initiate excision repair, effectively repairing CPDs [67]. Clinical trials have shown that topical application of T4N5 liposomes can prevent the development of new actinic keratoses and basal cell carcinomas in patients with XP; in addition, six-month follow-ups after the discontinuation of the medication showed that, unlike retinoids, there was no rebound increase in the rate of AKs and BCCs [68]. Nevertheless, this study was underpowered to analyze the rate of melanomas and SCCs [68]. Additionally, a murine model indicated that T4N5 application could serve as a useful adjunct to SC, reducing the harmful local effects of UVR such as sunburn cell formation [69].

### 2.3. 8-Oxoguanine Glycosylase

Oxidative stress resulting from the accumulation of ROS induces nucleotide oxidation and mutated forms, the most frequent being 8-oxo-7,8-dihydroguanine (8-oxoG) [70]. Guanine is a particularly susceptible base due to its relatively low oxidation threshold, and it is estimated that up to 3000 such mutations occur in each cell daily [71,72]. If 8-oxoG is not removed, it can result in the substitution of cytosine (C)-complementary to 8-oxoG-with adenine (A) during transcription, causing a G-C → A-T mutation [72].

To prevent this, evolutionarily conserved mechanisms have been extensively studied in bacterial models [73,74]. These are based on the NER pathway and are collectively referred to as the “guanine oxidation” (GO) system [75]. The three fundamental enzymes of this process (mutT, mutM, and mutY) were first described in bacterial models in 1992 [75], and their human equivalents have since been identified as Nudix hydrolase (NUDT1, also known as MutT human homolog 1, also named MTH1), 8-oxoG glycosylase (OGG1), and the adenine glycosylase MutY homolog (MUTYH) [76]. This oxidative damage repair system could play a crucial role in preventing skin cancer [77]. A reduction in OGG1 expression has been observed in basal cell carcinoma cells [78]. Another study found that while topical administration of OGG1 did not affect UVB-induced tumor multiplicity, it did reduce tumor size and significantly decrease tumor progression [79].

To our knowledge, no study has evaluated the isolated supplementation of OGG1. However, in 2015, Emanuele et al. [80] demonstrated the efficacy of a combination of traditional SPF50 SC with a liposome-encapsulated DNA repair enzyme complex (photolyase, endonuclease, and OGG1), and a potent antioxidant complex (carnosine, arazine, and ergothioneine). This study showed that the triple combination was more effective in reducing CPD, protein carbonylation, and the formation of 8-oxoG compared to SPF50 alone [80]. Notably, among the three markers, the formation of 8-oxoG exhibited the most modest changes, likely due to the high specificity of both the mutation and its repair process [80]. Similar results have been described in other publications where photolyase, endonuclease, and OGG1 have been combined [80].

## 3. Antioxidants

Antioxidant agents have become a major focus of interest in dermatology, not only in the field of photoprotection but also in inflammatory and immune-mediated dermatoses. Multiple substances have been studied for their antioxidant potential, both synthetic and natural. The latter have garnered significant interest, and an increasing number of plant-derived substances have been identified as potential antioxidants applicable in dermatology [81].

More than 50 plant species contain antioxidant substances, including well-known substances like flavonoids (found in species such as *Ginkgo biloba* and *Camellia sinensis*), vanillic acid, protocatechuic acid, caffeic acid, p-coumaric acid, ferulic acid, phenolic acids, tannins, stilbenes, carotenes, lycopene, lutein squalene, pycnogenol, and Polypodium leucotomos extract, among others (Table 5) [13].

### 3.1. Vitamins C and E

Vitamin E (α-tocopherol) and its derivatives (including γ- and δ-tocopherol) have demonstrated potent antioxidant effects at the cutaneous level by neutralizing ROS and potentially reducing UVR-induced damage [83]. These effects have been studied in vitro [84,85] and in murine models [83,86,87,88,89,90]. Their capacity to reduce UVA-induced immunosuppression in murine skin models has also been described [91,92].

Since the ability of vitamin E to scavenge ROS is based on its own oxidation, its combination with other antioxidant and stabilizing agents like vitamin C and ferulic acid may reduce the oxidation of vitamin E, thereby helping to preserve its antioxidant properties [93,94]. Some studies have compared the combination of vitamin E with vitamin C and ferulic acid to vitamin E and a placebo, demonstrating a greater ability of the combination to prevent skin carcinogenesis [95]. However, it is important to consider that application of vitamin E ester derivatives without stabilizing compounds has been associated with a possible increased risk of skin cancer, although these findings have not been confirmed [95,96].

Some population studies have shown a link between the consumption of vitamins E and C and a lower incidence of skin cancer; however, given the characteristics of the studies and the their limited sample size, these results should be interpreted with caution [97,98]. In human skin, the topical application of vitamins C and E has been shown to increase the MED in healthy subjects [99]. Similarly, other studies have confirmed this increase in the MED, as well as a decrease in sunburn cells, thymine dimers, and p53 expression [100,101]. A lower expression of pro-inflammatory cytokine RNA has also been confirmed [100,101].

### 3.2. Polypodium Leucotomos

#### 3.2.1. Biological Origin and Mechanism of Action

Polypodium leucotomos (PL) is a tropical fern belonging to the family Polypodiaceae [102]. PL hydrophilic extract (PLE), obtained from the aerial parts of the plant, is rich in phenolic compounds endowed with strong antioxidant and photoprotective properties [103,104,105]. Caffeic and ferulic acid are the most relevant [103,104,105]. 

The effects of these phenolic constituents on cellular homeostasis have been demonstrated both in vivo and in vitro, and they include the following [106,107,108,109,110]:

- Direct absorption of UV photons, preventing the formation of photoproducts;
- Scavenging of reactive oxygen (ROS) and nitrogen species, mitigating photo-oxidative stress;
- Prevention of lipid and glutathione peroxidation;
- Reduction of cellular proliferation;
- Prevention of Langerhans cell (LC) abrogation;
- Preservation of skin immune surveillance.

All of these combined limit UV-induced erythema, skin aging, and carcinogenesis [111]. For these reasons, PLE was the first systemic photoprotective agent (SPA) with well-documented effects in humans [109]. SPAs offer several advantages compared to classical topical SCs: ease of use, better real-life adherence, and uniform photoprotection on the total body surface [112]. 

#### 3.2.2. Experimental and Clinical Evidence

Prior to its widespread commercial marketing, PLE had been used for centuries in Central American folklore medicine to relieve different skin conditions [113]. Due to its immunomodulatory properties, PLE has been tried in the management of inflammatory dermatoses, including vitiligo, psoriasis, and atopic dermatitis, with varying results [114,115,116,117,118]. Since these are out of reach of our work, we only reviewed clinical and experimental trials on the use of PLE as an SPA (Table 6). 

Five out of six studies were RCTs with proper sample sizes [109,119,120,121,122]. Follow-up periods were highly variable and ranged from 2 days to 12 months [104,109,122]. Clinical indications significantly differed between studies, from young healthy individuals (n = 3) [104,109,119] to patients with AK (n = 2) [121,122] and melasma (n = 1) [120]. Most subjects were phototypes II or III [104,119,121,122]. Both topical and systemic administrations of PLE were investigated. Oral doses fluctuated, ranging from 240 mg/day to 960 mg/day [121,122]. The selected anatomical sites varied according to the characteristics of the recruited population: non-exposed (healthy individuals) [109,116,119] or sun-exposed sites (melasma and AK) [120,121,122]. The types of assessment were considerably diverse, including biopsies [109,119] and non-invasive imaging techniques such as spectrophotometry and dermoscopy of RCM [120,121,122]. 

Gonzalez et al. [109] conducted the first clinical trial of PLE as an SPA in humans (Table 6). They assessed whether PLE could protect the human skin from UV-induced erythema in vivo. Individuals were instructed to expose the tested areas to natural solar radiation in Malaga (Andalucia, Spain, 36°45′ N, 4°25′ O) for up to 120 min in August between 11:00 a.m. and 2:00 p.m. [109]. An increment in the mean time for eliciting erythema was detected in both non-photosensitized individuals (80 min in the topical PLE group and 90 min in the oral PLE group, *p* < 0.05) [109]. Photosensitized individuals with either 5-methoxypsoralen (5-MOP) or 8-methoxypsoralen (8-MOP) and who were treated with oral PLE had increased MPDs (the minimal erythema dose required to induce a phototoxic reaction 72 h after UVA radiation): 45 vs. 18 ± 4.1 and 57.5 ± 11.2 vs. 7.5, respectively [109]. Interestingly, MPDs could not be measured in skin sites pretreated with SPF15 SC and a 10% PLE solution, as no phototoxic reaction was elicited due to complete protection (CP). Despite their astounding outcomes and the confirmation of the antioxidant properties of PLE in vivo, their interpretation of their findings was limited because the erythematous and tanning reactions were only clinically assessed by two investigators and not by means of objective instruments such as chromatography. 

Nevertheless, these outcomes were later confirmed in an open-label prospective trial conducted by Middelkamp et al. (Table 6) [104]. Twelve healthy young subjects with low phototypes (I–II) were recruited [104]. Seven back sites were selected and twice exposed to 1 MED (n = 5) and 2–3 MED radiations (n = 2) [104]. Only before the second exposure, patients were pretreated with oral PLE (7.5 mg/kg) 30, 60, 90, 120, and 180 min before UVR [104]. Erythema was assessed 24 h later [104]. Patients treated with PLE showed statistically significant less erythema (*p* < 0.01), although the difference was lost after 3 h [104]. This indicates that oral PLE should be readministered at least once more per day to achieve adequate sun protection during the hours with the highest UV index [104].

PLE has also been confirmed to limit UV-induced cutaneous hyperpigmentation [109]. Gonzalez et al. [109] reported that non-photosensitized subjects showed increased IPD (the minimal UV dose required to induce darkening pigment reaction 30–45 min after exposure) if treated with 10% PLE lotion (56 ± 16.73) and oral PLE (75 ± 17.32) when compared to the placebo (25.9 ± 10.62) and this was similar to the IPD present in SPF15 SC-treated sites (80 ± 14.14) [109]. IPD could not be calculated in areas exposed to 25–50% PLE lotions, as they offered complete protection [109]. 

The delayed tanning reaction was also assessed as the MMD (minimal melanogenic dose required for a delayed hyperpigmentation 5 days after exposure) [109]. The MMD could not be measured in areas treated with SPF15 SC or 25–50% PLE lotions, since they achieved complete protection [109]. In non-photosensitized individuals, no statistically significant differences (*p* > 0.05) were found between 10% PLE lotion (88 ± 10.95) and oral PLE (82.5 ± 25.9) when compared to the placebo (72 ± 22.8) [109]. The authors considered that these outcomes confirmed that PLE could not appropriately filter UVR [109]. 

These outcomes were remarkable, as PLE could have served as an excellent photoprotective agent in the prevention of post-inflammatory hyperpigmentation in high-phototype patients. Sixteen years later, Ahmed et al. [120] conducted the first RCT on the efficacy of oral PLE in the management of moderate-to-severe facial melasma in non-pregnant and non-lactating Hispanic women with a melanin index ≥30 (Table 6) [120]. A regimen of 240 mg/8 h for 3 months was chosen. Patients in both groups were instructed to use standard broad-spectrum SC once daily [120]. At the 3-month follow-up visit, the melanin index measured by narrowband spectrophotometry improved in both groups (−28.8% vs. −13.8%) compared to their baseline scores, although no statistically significant differences were found between them either in severity scores (MASI, *p* = 0.62) or in improvement of quality of life (MELASQoL) after treatment [120].

One RCT showed that PLE can reduce the incidence of new scalp AKs after two sessions of MAL-PDT (3-h incubation, λ = 635 nm, 37 J/cm^2^, 8 min, 1 week apart) [121]. AK severity was at least moderate, since recruited patients in both groups still displayed a higher basal count (7–8), even with sizes larger than 1 cm (n = 16, 47.06%) in spite of the field cancerization treatment received 1 week before the onset of PLE exposure [121]. Subjects were randomized to receive oral PLE (960 mg/24 h in the first month, 480 mg/24 h from the second to the six month) or a placebo [121]. All were instructed to use SPF50+ SC twice a day during sun exposure (Table 6). At the 6-month follow-up visit, those treated with oral PLE showed a similar final AK count (1 vs. 2, *p* = 0.409) but experienced a greater long-term clearance of AK lesions (−8 vs. −3, *p* = 0.04) [121]. Apart from MAL-PDT, the authors did not specify if patients had been previously managed with field cancerization treatments [121]. 

The most recent study on the use of PLE as an SPA has been published in 2023 by Pellacani et al. [122]. This trial has several advantages that guarantee the excellent validity and extrapolation of the results [122]. Firstly, they compared the use of PLE with SPF100 SC ad libitum, as in the real-life setting [122]. The study had a considerably long follow-up period (12 months) and a large sample size (n = 131), which is notable compared to previous clinical trials involving AK patients [122]. To the best of our knowledge, it is the only trial with PLE that recruited patients with a personal history of NMSC (n = 47, 35.9%) [122]. They also counted lesions in non-head and -neck areas, which are excluded from most AK trials [122]. Patients were randomly assigned to three groups: placebo, topical PLE gel, and topical PLE gel + oral PLE (240 mg/24 h). At the 6-month follow-up visit, subjects treated with topical PLE alone or topical + oral PLE showed a lower incidence of new AKs (1 vs. 0 vs. 10, *p* = 0.008) and a lower need of field-cancerization-targeted treatments (10.3% vs. 2.9% vs. 23.1%, *p* = 0.027) as compared to the placebo (Table 6) [122]. However, no statistically significant differences were detected in both parameters at the 12-month follow-up visit (0 vs. 0 vs. 4, *p* = 0.054/9.1% vs. 0% vs. 13.3%, *p* = 0.112). At this point, treatment with PLE only showed significant benefits consisting of less clinical hyperkeratosis (5.9% vs. 3% vs. 30%, *p* = 0.002) and partial normalization of honeycomb pattern with RCM (45% vs. 50% vs. 26%, *p* = 0.04), without improvement of the involved surface area (*p* = 0.614) and the AKASI (3.3 ± 1.2 vs. 3.1 ± 1.1 vs. 3.5 ± 1.3, *p* = 0.427) and IGA (*p* = 0.105) scores. 

These two trials highlight the efficacy of topical and oral PLE in preventing the long-term appearance of new AK lesions, reducing the need for field-cancerization-targeted treatments [121,122]. This is of the utmost importance in the clinical setting, as severely photodamaged individuals may be hesitant to undergo these therapies due to concerns about potential side effects [123].

Apart from the previously mentioned clinical findings, histological data *in vivo* sup-port the use of PLE as a photoprotective agent in humans [104,109,119]. Following the UVR equivalent to 2–3 MEDs, oral PLE has been shown to significantly reduce the sunburn reaction, decrease the density of papillary mastocytes (126.4/mm^2^ vs. 173.76/mm^2^, *p* ≤ 0.05) and sunburn cells (16.3/mm^2^ vs. 22.4/mm^2^, *p* = 0.03), reduce the formation of CPD (CPD positive cells: 43.7 vs. 74.7, *p* < 0.001), and limit the mitotic proliferation of keratinocytes (Ki67+: 25.94% vs. 38.85%, *p* < 0.001) [104,109]. Despite its efficacy in other aspects, UVA-induced damage of mitochondrial DNA does not seem to be mitigated by oral supplementation of PLE, as no statistically significant difference has been found in the rate of “common deletion” (CD) after 2–3 MED-A radiation (*p* = 0.06) [119]. However, a low sample size (n = 10) and the simultaneous use of SC ad libitum could have affected the power of this specific study [119]. 

Regarding the preservation of immunosurveillance, Gonzalez et al. [109] confirmed that topical and oral PLE were the only agents which preserved the dendritic morphology of LCs when compared to placebo and SPF15+ SC. Additionally, systemic administration of PLE completely prevented the depletion of LCs after midday summer sun radiation (Table 6) [109]. Nevertheless, this outcome was not replicated by Middelkamp et al. [104], as no difference in LC density was detected when compared to the placebo (24.8/mm^2^ vs. 18.56/mm^2^, *p* > 0.05). 

Notwithstanding these promising results, several biases and characteristics of the studies necessitate cautious interpretation. As only two trials compared the use of PLE with standard photoprotection, there are currently no data available on its relative efficacy compared to commonly used SCs [109,122]. This is crucial in the clinical setting, as dermatologists and other physicians need, on a daily basis, to determine the most appropriate SC for each patient based on its efficacy, tolerance, safety, and expected adherence. Additionally, it would be interesting to specifically investigate whether the simultaneous use of oral PLE with other topical SCs has a synergistic effect in the prevention of AKs and skin cancer, especially in severely photodamaged or high-risk individuals. 

Special consideration should be given to the moderate dropout ratio (approximately 15%) detected in three of the trials, which could hint at a potentially improvable compliance in patients with longer treatment durations (3–12 months) [120,121,122]. For these reasons, it would also be interesting to assess whether the adherence of real-life patients could be improved if the use of PLE was selectively encouraged in seasons or places with a higher UV index. 

On the other hand, since immunosuppressed individuals were excluded from all trials, their outcomes cannot be extrapolated to transplanted patients, which are precisely one of the population subsets at higher risk of developing NMSC and are thus in dire need of strong and persistent photoprotective agents. 

Information is still lacking on the overall safety of topical and oral PLE. Only in one study was this concern explicitly addressed, although in a vague manner without specific outcomes [120]. Indirect observations may suggest oral PLE has an adequate tolerability, as it increased the adherence to overall photoprotection habits in AK patients [122]. 

### 3.3. Green Tea Polyphenols

Tea, the second most popular beverage worldwide after water, is obtained from the fresh leaves and buds of the plant *Camellia sinensis* [124,125]. Depending on the oxidation and polymerisation state of its polyphenolic compounds, it is commercially available in three different forms: black tea, green tea (GT), and oolong tea [124].

Recently, botanical supplements such as GT have garnered considerable interest among researchers and consumers due to their antioxidant, photoprotective, and anti-inflammatory effects [124,126]. Epidemiological studies have hinted at a link between GT consumption and a lower risk of developing cancers of the esophagus, oropharynx, stomach, bladder, liver, and urinary tract [124,125,127]. 

GT is rich in epicatechins, such as (-)-epicatechin, (-)-epicatechin-3-gallate, (-)-epigallocatechin, and (-)-epigallocatechin-3-gallate (EGCG) [124,125]. These, especially EGCG, are considerably more antioxidant than theaflavins and thearubigins, the main constituents of black tea [124,125]. 

The initial evidence of the photoprotective effects of GT was reported by Wang et al. [128] in 1991. SKH-1 hairless mice, which were topically or systemically administered GT extracts, showed a dose-dependent prolongation in the mean time for developing UVB-induced sunburn reaction, skin cancer, and immunosuppression [128].

To date, three experimental trials on the photoprotective effects of green tea polyphenols (GTPs) on human skin have been published (Table 7) [17,129,130].

The methodology was similar among them: GTPs were topically administered for 15–30 min on non-exposed sites (buttocks and back) before solar-simulated UVB radiation (0.5–4 MEDs) [17,129,130]. The acute clinical and histological effects were assessed with a chromameter and biopsies 1–3 days after irradiation [17,129,130].

GTPs have been shown to protect from sunburns, especially UVB-induced erythema [17,129]. Katiyar et al. [17] first showed that pretreatment with GTPs significantly reduced the appearance of erythema (−84%). This was later confirmed by Elmets et al. [129], who observed that GTPs acted in a dose-dependent manner. The 10% GTP solution offered almost complete protection 48 and 72 h after the initial radiation (Table 7) [129].

GTPs prevent UV-induced DNA damage and immunosuppression in vivo. Sites pretreated with GTP cream displayed a remarkably lower percentage of epidermal CPD-positive cells (25% vs. 80%, −60%, *p* < 0.0005) and less 8-OHdG 1 day after UVB radiation (4 MEDs) [17,130]. This finding was later replicated by Elmets et al. [129] Analyzing the biopsies taken 24 h after UVB radiation (2 MEDs) of sites pretreated with 1–10% GTP solution, they found less sunburn cells (1.9 ± 0.3/mm^2^ vs. 5.8 ± 0.7/mm^2^, *p* < 0.01), a higher density of LCs (377 ± 28/mm^2^ vs. 88 ± 29/mm^2^, *p* < 0.01), and reduced epidermal DNA damage detected through ^32^P labelling [129].

Camouse et al. [130] detected, in biopsies taken 24 h after UVB radiation (2 MEDs), that GTPs partially protected against LC depletion (−35% vs. −57%, *p* = 0.03), although no statistically significant differences were found when compared to white tea (WT) (*p* = 0.09). 

Despite promising results, these three studies show remarkable limitations that require careful and thorough interpretation. Most had a low sample size (n < 10), follow-up periods were extremely short (1–3 days), and did not consider several factors (clinical indications, adherence, type of vehicle, safety) which are important in real-life skin photoprotection.

Unfortunately, the only clinical response addressed in these studies was UV-induced erythema. For this reason, nothing is known about the possible impact of GTPs on photoaging (colour, wrinkles, texture) and different clinical indications (such as AKs). 

Since only healthy subjects were recruited, the effects of GTPs on patients with a personal history of cancer or moderate-to-severe photodamage or those under immunosuppression still need to be elucidated. 

Considering all of the limitations mentioned, clinical evidence of GTPs’ photoprotective effects is still limited for recommending their use in the general population. RCTs with larger sample sizes and longer follow-up periods are thus needed to discern whether GTPs prevent the development of cutaneous premalignant and malignant disorders. 

### 3.4. Punica granatum

An extract of *Punica granatum* (*Punicaceae*) seeds has been studied. An investigation conducted by Kaur and Saraf [131] showed an improvement in facial skin mechanical (viscoelasticity) and biochemical parameters (catalase and ascorbic acid concentration) when applying an ethanolic extract of *Punica granatum* through nanotransfersomes. In addition, a reduction in malondialdehyde levels was also noted [131]. These results showed the antiaging effect of the nanotransfersome-loaded cream [131]. The investigators also compared the effect of *Punica granatum* through various formulations, obtaining the following order: nanotransfersomal cream > conventional cream > blank nanotransfersomal cream > base cream [132]. The observed antiaging effect was pointed out as a feature of antioxidants such as anthocyanins, ellagic acid, and hydrolysable tannins present in the extract [132].

### 3.5. Resveratrol

Resveratrol is a naturally occurring polyphenolic compound with strong antioxidant properties [133,134]. It is abundantly found in nuts, grapes, berries, and red wine [133,134]. Jang et al. [135] firstly reported the chemopreventive property of resveratrol in 1997. Aziz et al. [133] studied the protective effect of resveratrol against chronic UVB exposure-mediated damages to SKH-1 hairless mouse skin [133]. The data from this study demonstrated that topical application of resveratrol to mouse skin (both pre- and post-UVB treatment) resulted in a highly significant inhibition in tumor incidence and multiplicity as well as a delay in the onset of tumorigenesis [133]. Another study of the same group showed that resveratrol protection against UVB-mediated damages to mouse skin is based on the apoptotic elimination of damaged cells via an inhibition of the survivin pathway [136].

### 3.6. Forskolin

Forskolin is a naturally derived diterpenoid extracted from the roots of the Plectranthus barbatus (*Coleus forskolii*) plant that grows in Asia and that has long been traditionally used in teas and therapeutic preparations [137]. Forskolin, which is a skin-permeable compound, directly activates adenylate cyclase to induce production of cAMP [137]. Pharmacologic stimulation of cAMP using forskolin may protect the skin in multiple ways [137]. Firstly, it has shown the capacity of inducing melanin production. Moreover, in the existing literature, cAMP has proven to enhance keratinocyte migration to promote wound healing [138] and to decrease blister formation [139]. Other studies reported that forskolin protects against oxidative stress generation by decreasing nitric oxide levels [140] and enhancing stimulation of the cytoplasmic antioxidant enzyme copper/zinc superoxide dismutase (Cu/ZnSOD) [141].

## 4. Conclusions

The exploration of “active photoprotection” through the utilization of DNA-repair enzymes and naturally occurring antioxidant molecules represents a significant advancement in sun protection research. Our review underscores the importance of moving beyond conventional approaches towards interventions capable of not only preventing but also partially reversing UV-induced damage.

Through an analysis of state-of-the-art research, including clinical trials and in vivo models, it becomes evident that photolyases, *Polypodium leucotomos* extract, and other bioactive compounds offer multifaceted benefits in safeguarding skin health, reducing the risk of mutagenesis, carcinogenesis, and premature aging. Their synergistic effects highlight the potential for combination therapies. Continued research into their mechanisms of action, optimal formulations, and clinical efficacy is needed for confirming these findings. 

## Figures and Tables

**Table 1:** Use of topical photolyases on healthy human skin: experimental trials.

	Stege et al. [33]	Berardesca et al. [34]
STUDY DESIGN		
Year of publication	2000	2011
Type of study	Experimental trial	Pilot interventional clinical study
n	19	10
UVR	UVB: 1–2 MEDs	Solar-simulated UVR (ssUVR) (λ = 290–400 nm): 3 MEDs daily for 4 consecutive days.
PHOTOLYASES	*Anacystis nidulans* 1% (liposomes)*:* Photosome Daytime Formula^®^ (hidrogel)	*Anacystis nidulans* 1% (liposomes)
RESULTS	TPHs reduced the formation of CPD in a time-dependent manner and partially reversed UVB–induced immunosuppression	TPH + SC significantly decreased the levels of CPD (−93%) and cellular apoptosis (−82%) and were superior to SC alone (*p* < 0.001)

**Table 2:** Use of topical photolyases for the management of AK in special situations: for XP, on forearms, and after MAL-PDT treatment.

	Giustini et al. [40]	Eibenschutz et al. [44]	Alvares et al. [47]
STUDY DESIGN			
Year of publication	2014	2016	2022
Type of study	Retrospective case study	Randomized assessor-blinded parallel comparative trial	Double-blinded RCT
n	8	30 (1:1)	40 (1:1)
Control group	None	SC (SPF50+)	SC (SPF99)
Follow-up	1 year	9 months	2 months
PHOTOLYASE	Eryfotona AK-NMSC fluid^®^ (ISDIN, Barcelona, Spain)	Eryfotona AK-NMSC fluid^®^ (ISDIN, Barcelona, Spain)	Eryfotona AK-NMSC fluid^®^ (ISDIN, Barcelona, Spain)
CLINICAL INDICATION	XP	AK	AK
Anatomical location	Unspecified	Face and/or scalp	Forearms
Previous field-cancerization targeted treatments	Unspecified	MAL-PDT (λ = 630 nm, 37 J/cm^2^) 2 weeks before	None in the previous 6 months
Baseline AK count	14	2 ± 2 vs. 0.6 ± 0.5, *p* > 0.05	7 (6–9)
RESULTS	TPH reduced the incidence of new AK (−64.29%), BCC (−56%), and SCC (−100%) after 1 year of treatment	Eryfotona^®^-treated patients showed a lower final AK count (1 ± 1.1 vs. 3.6 ± 3.8, *p* < 0.01) and less need of field-targeted therapies (0% vs. 67%, *p* < 0.01).	TPH + SC failed to prove superiority to SC alone

**Table 3:** Use of topical photolyases in AK management: studies based on imaging techniques.

	Puig et al. [39]	Rstom et al. [41]	Carducci et al. [42]	Laino et al. [43]	Puviani et al. [37]	Moscarella et al. [45]
STUDY DESIGN						
Year of publication	2014	2014	2015	2015	2015	2017
Type of study	Pilot prospective controlled interventional clinical study	Longitudinal observational clinical trial	RCT	Prospective cohort	Pilot prospective open-label study	Double-blinded controlled randomized pilot study
n	13 (3:1)	14	28 (1:1)	30	11	50 (24:26)
Control group	SC	None	SC SPF50	None	None	SC SPF50+
Follow-up	1 month	3 months	6 months	9 months	3 months	6 months
PHOTOLYASE	Eryfotona AK-NMSC fluid^®^ (ISDIN, Barcelona, Spain)	Eryfotona AK-NMSC fluid^®^ (ISDIN, Barcelona, Spain)	1% photolyase (*Anacystis nidulans*) + 1% endonuclease (*Micrococcus luteus*)	Eryfotona AK-NMSC fluid^®^ (ISDIN, Barcelona, Spain)	Eryfotona AK-NMSC fluid^®^ (ISDIN, Barcelona, Spain)	Eryfotona AK-NMSC fluid^®^ (ISDIN, Barcelona, Spain)
AK						
Anatomical location	Sun-exposed areas, tested sites’ size > 12.96 cm^2^	Face	Face and scalp	Scalp	Unspecified: “usually the face and scalp”	Face and scalp
IMAGING TECHNIQUE	Dermoscopy and RCM	Dermoscopy and RCM	Fluorescence	Active telethermography (ATT)	Colorimetry	Dermoscopy and RCM
RESULTS	Improvement only noted in patients treated with Eryfotona^®^, with less erythema (*p* = 0.03), scaling (*p* = 0.028), coherence of corneocytes (*p* = 0.018), atypical honeycomb pattern (*p* = 0.0005), and round nucleated cells at the *stratum granulosum* (*p* = 0.019)	Improvement only noted in grade I lesions: less erythema, scaling, and atypical honeycomb pattern	The combination of photolyases and endonucleases was superior to SC alone in reducing the cancerization field fluorescence (*p* < 0.001)	Decreased HH size (−82.37%) and increased TRT	Less hemoglobin (−34%, *p* = 0.0124) detected in the targeted lesion after treatment	Improved dermoscopic parameters (erythema, pigmentation, follicular plugs) compared to SC alone. No differences between groups were found with RCM.

**Table 4:** Use of topical photolyases in the real-life management of AK.

	Puviani et al. [36]	Vañó Galván et al. [48]	Navarrete-Dechent et al. [46]
STUDY DESIGN			
Year of publication	2013	2016	2017
n	6	41	9
Follow-up	1.5–2 months	6 months	3 months
PHOTOLYASE	Eryfotona AK-NMSC fluid^®^ (ISDIN, Barcelona, Spain)	Eryfotona AK-NMSC fluid^®^ (ISDIN, Barcelona, Spain)	Eryfotona AK-NMSC fluid^®^ (ISDIN, Barcelona, Spain)
CLINICAL INDICATION	AK	AK (treatable with cryotherapy)	AK
Anatomical location	Face and scalp	Scalp (33, 80%), face (8, 20%)	Face, scalp, hands, and forearms
Baseline AK count	11.67 (1–25)	9.56	Unspecified: “multiple”
PATIENTS’ BASELINE CHARACTERISTICS AND ELEGIBILITY CRITERIA			
Age (years)	67.5 (65–74)	75.3 (58–85)	70.6
Male	6 (100%)	37 (90.24%)	5 (55.56%)
Immunosuppressed	0	Unspecified	2 (22.22%)
*Xeroderma pigmentosum*	0	Unspecified	Unspecified
Personal history of NMSC	1 (16.67%)	Unspecified	Unspecified
CLINICAL RESULTS			
RESULTS	All patients (100%) achieved a clearance rate greater than 50%	Dramatic reduction in AK count (−84.21%). All patients (100%) achieved a clearance rate greater than 50%	Lower final AK count (−76.6%, *p* < 0.0001). All patients (100%) achieved a clearance rate greater than 50%

**Table 5:** Well-known plant-derived antioxidants used in photoprotection [13,81,82].

Flavonoids	Phenolic Acids	Other
Quercetin	p-coumaric acid	Vitamins C and E
Rutin	Ferulic acid	
Kaempferol		*Polypodium leucotomos* extract
Isorhamnetin		*Punica granaticum*
Apigenin		Resveratrol
Luteonin		Forskolin
Hespertin		Tannins: green tea polyphenols
Cyanidine		Carotenes
Peonidin		Licopenes
		Stilbenes
		Lutein

**Table 6:** Effects of PLE on human skin: in vivo trials.

	Gonzalez et al. [109]	Middelkamp-Hup et al. [104]	Villa et al. [119]	Ahmed et al. [120]	Auriemma et al. [121]	Pellacani et al. [122]
STUDY DESIGN						
Year of publication	1997	2004	2010	2013	2014	2023
Type of study	RCT	Open-label prospective trial	Randomized investigator-blinded trial	RCT	RCT	Open-label assessor-blinded RCT
n	21 (8:13)	9	10 (5:5)	40 (20:20)	40 (20:20)	131 (43 SC: 44 topical PLE (T): 44 topical and oral PLE (TO))
Follow-up	2 days	12 months	1 week	4 months	6 months	12 months
UV RADIATION	Midday solar ultraviolet radiation (sUVR) in Malaga (Andalucia, Spain, 36°45′ N, 4°25′ O) in August, between 11:00 a.m. and 2:00 p.m. Patients were exposed for up to 120 min.	5 sites (MED), 2 sites (2–3 MEDs)	2–3 MED-A	Regular sun exposure	Regular sun exposure	Regular sun exposure
***POLYPODIUM LEUCOTOMOS* EXTRACT**						
Composition	*Polypodium leucotomos* (DIFUR^®^, Industrial Farmaceutica Cantabria, SA, Madrid, Spain).	*Polypodium leucotomos* (Fernblock^®^, Industrial Farmaceutica Cantabria, SA, Madrid, Spain).	Unspecified	Unspecified	*Polypodium leucotomos* (Fernblock^®^, Industrial Farmaceutica Cantabria, SA, Madrid, Spain).	*Polypodium leucotomos* (Fernblock^®^, Industrial Farmaceutica Cantabria, SA, Madrid, Spain).
Route of administration	Topical or oral (groups B or D	Oral	Oral	Oral	Oral	Topical and topical + oral
Posology	Topical: 15–30 min before sUVR. Oral: 1 day before sUVR: 240 mg/8 h. Day of exposure: 240 mg 3 h before sUVR.	First dose: 7.5 mg/kg, evening before the second UVR Second dose: 7.5 mg/kg, 30 min–3 h before second UVR.	240 mg 8 and 2 h before UVR	240 mg/8 h, 12 weeks	First month: 960 mg/24 h 2nd–6th months: 480 mg/24 h	Topical: twice daily Oral: 240 mg/24 h
ADDITIONAL SUNSCREEN (SPF)	15	None	None	55	50+	100+
CLINICAL INDICATION	Healthy individuals	Healthy individuals	Healthy individuals	Facial melasma (moderate-to-severe)	AK	AK
CLINICAL RESULTS	Increased time to elicit erythema and phototoxic reactions	Less erythema was observed in patients treated with oral PLE up to 120 min post-UVR (*p* < 0.01)	Unassessed	Improvement of MASI, without statistical significance compared to SC alone (*p* = 0.62)	Higher clearance rate compared to the placebo (−81% vs. −71%, *p* = 0.04)	Lower incidence of new AK lesions (*p* = 0.008) and need of field-cancerization therapies (*p* = 0.027) after 6 months of treatment
HISTOLOGICAL AND RCM RESULTS	Oral PLE prevented the depletion of LCs	Reduced density of sunburn cells (*p* = 0.03) and papillary dermal mast cells (*p* ≤ 0.05). Lower CPD levels (*p* < 0.001) and cellular proliferation (*p* < 0.001)	PLE was not superior in reducing UV-induced mitochondrial DNA mutations	Unassessed	Unassessed	PLE was superior in reducing the honeycomb pattern (*p* = 0.04) after 1 year of treatment

**Table 7:** Green tea polyphenols: experimental trials in humans.

	Katiyar et al. [17]	Elmets et al. [129]	Camouse et al. [130]
STUDY DESIGN			
Year of publication	2000	2001	2009
Type of study	Experimental trial	Experimental trial	Double-blinded experimental trial
n	6	6	90
UV RADIATION	UVB: 0.5, 1, 2 and 4 MEDs	ssUVR 2 MEDs UVA radiation (135 J/cm^2^)	ssUVR (0.75 and 2 MEDs)
GREEN TEA POLYPHENOLS			
Composition	Epicatechin (6%), epigallocatechin (5%), epigallocatechin-3-gallate (65%), epicatechin-3-gallate (24%). Different amounts: 3 mg/2.5 cm^2^ (remaining), 1–4 mg (4 MEDs)	A 1–10% GTP solution (200 mL, ethanol/water vehicle): >95% (epicatechin, epigallocatechin-3-gallate, epigallocatechin-3-gallate). Mitsui Norin^®^, Shizuoka, Japan	Unspecified
CLINICAL INDICATION	Healthy individuals	Healthy individuals	Healthy individuals
CLINICAL RESULTS	Pretreatment with GTP protected from UVB-induced erythema appearance (−84%)	The GTP solution reduced erythema in a dose-dependent manner. The 10% GTP solution achieved almost complete protection 48 and 72 h after ssUVR (*p* < 0.01)	Unassessed
HISTOLOGICAL RESULTS	GTP partially protected cells from the deleterious effects of UVB radiation, reducing the levels of CPD (−60%, *p* < 0.0005) in a dose-dependent manner.	Reduced density of sunburn cells and DNA damage in GTP-exposed participants (−68%, *p* < 0.01). GTPs partially reversed UV-induced depletion of LC (*p* < 0.01)	WT and GT similarly prevented the depletion of LCs after UVR (−22% vs. −35%, *p* = 0.09) and reduced the formation of 8-OHdG

## Abbreviations

5-MOP	5-methoxypsoralen
8-MOP	8-methoxypsoralen
8-oxoG	8-oxo-7,8-dihydroguanine
A	adenine
AK	actinic keratosis
AKSS	actinic keratosis severity score
ATT	active telethermography
au	arbitrary units
BCC	basal cell carcinoma
BSI	baseline severity index
C	cytosine
CD	common deletion
CP	complete protection
CPD	cyclobutane pyrimidine dimer
Cu/ZnSOD	copper/zinc superoxide dismutase
FF	Fitzpatrick’s phototype
FPS	forearm photoaging scale
GO	guanine oxidation
GT	green tea
HH	hyperthermic halo
IGII	investigator global improvement index
LC	Langerhans cells
MED	minimal erythema dose
MTH1	MutT human homolog 1
MUTYH	adenine glycosylase MutY homolog
NER	nucleotide excision repair
NMSC	nonmelanoma skin cancer
NUDT1	Nudix hydrolase
nPh	non-photosensitized
OGG1	8-oxoguanine glycosylase
PDT	photodynamic therapy
P	placebo
Ph	photosensitized
PoLE	polymorphic light eruption
RCM	reflectance confocal microscopy
RCT	randomized clinical trial
ROS	reactive oxygen species
SC	sunscreen
SCC	squamous cell carcinoma
SPA	systemic photoprotective agent
SPF	sun protection factor
sUVR	solar ultraviolet radiation
ssUVR	solar-simulated ultraviolet radiation
T	topical PLE
T4N5	T4 endonuclease V
TCS	total clinical score
TO	topical and oral PLE
TPH	topical photolyases
TRT	thermal recovery time
UV	ultraviolet
UVR	ultraviolet radiation
V	vehicle
VL	visible light
T0	baseline visit
T1	first follow-up visit
WT	white tea
XP	xeroderma pigmentosum
